# Purkinje Cell Signaling Deficits in Animal Models of Ataxia

**DOI:** 10.3389/fnsyn.2018.00006

**Published:** 2018-04-26

**Authors:** Eriola Hoxha, Ilaria Balbo, Maria Concetta Miniaci, Filippo Tempia

**Affiliations:** ^1^Neuroscience Institute Cavalieri Ottolenghi (NICO), Turin, Italy; ^2^Department of Neuroscience, University of Torino, Turin, Italy; ^3^Department of Pharmacy, School of Medicine, University of Naples Federico II, Naples, Italy; ^4^National Institute of Neuroscience (INN), Turin, Italy

**Keywords:** cerebellum, ataxia, Purkinje cell, ion channels, firing, parallel fiber, climbing fiber

## Abstract

Purkinje cell (PC) dysfunction or degeneration is the most frequent finding in animal models with ataxic symptoms. Mutations affecting intrinsic membrane properties can lead to ataxia by altering the firing rate of PCs or their firing pattern. However, the relationship between specific firing alterations and motor symptoms is not yet clear, and in some cases PC dysfunction precedes the onset of ataxic signs. Moreover, a great variety of ionic and synaptic mechanisms can affect PC signaling, resulting in different features of motor dysfunction. Mutations affecting Na^+^ channels (Na_V_1.1, Na_V_1.6, Na_V_β4, Fgf14 or Rer1) reduce the firing rate of PCs, mainly via an impairment of the Na^+^ resurgent current. Mutations that reduce Kv3 currents limit the firing rate frequency range. Mutations of Kv1 channels act mainly on inhibitory interneurons, generating excessive GABAergic signaling onto PCs, resulting in episodic ataxia. Kv4.3 mutations are responsible for a complex syndrome with several neurologic dysfunctions including ataxia. Mutations of either Cav or BK channels have similar consequences, consisting in a disruption of the firing pattern of PCs, with loss of precision, leading to ataxia. Another category of pathogenic mechanisms of ataxia regards alterations of synaptic signals arriving at the PC. At the parallel fiber (PF)-PC synapse, mutations of glutamate delta-2 (GluD2) or its ligand Crbl1 are responsible for the loss of synaptic contacts, abolishment of long-term depression (LTD) and motor deficits. At the same synapse, a correct function of metabotropic glutamate receptor 1 (mGlu1) receptors is necessary to avoid ataxia. Failure of climbing fiber (CF) maturation and establishment of PC mono-innervation occurs in a great number of mutant mice, including mGlu1 and its transduction pathway, GluD2, semaphorins and their receptors. All these models have in common the alteration of PC output signals, due to a variety of mechanisms affecting incoming synaptic signals or the way they are processed by the repertoire of ionic channels responsible for intrinsic membrane properties. Although the PC is a final common pathway of ataxia, the link between specific firing alterations and neurologic symptoms has not yet been systematically studied and the alterations of the cerebellar contribution to motor signals are still unknown.

## Introduction

In this review, we focus on the molecular and cellular mechanisms responsible for the alterations of basic physiological functions of Purkinje cells (PCs), including intrinsic membrane properties and synaptic signaling, commonly observed in animal models of ataxia. The PC output is generated by processing of incoming synaptic signals, which reach the cerebellar cortex by two pathways: the mossy fiber-granule cell-parallel fiber (PF) pathway and the climbing fibers (CFs) contacting directly PCs. Several studies indicate that both systems can be shaped by sensory experience and neuromodulation (Ito, 1984; Lippiello et al., 2015, 2016). Synaptic plastic changes in the cerebellar circuit are thought to underlie motor learning and behavioral control.

**Figure 1:**
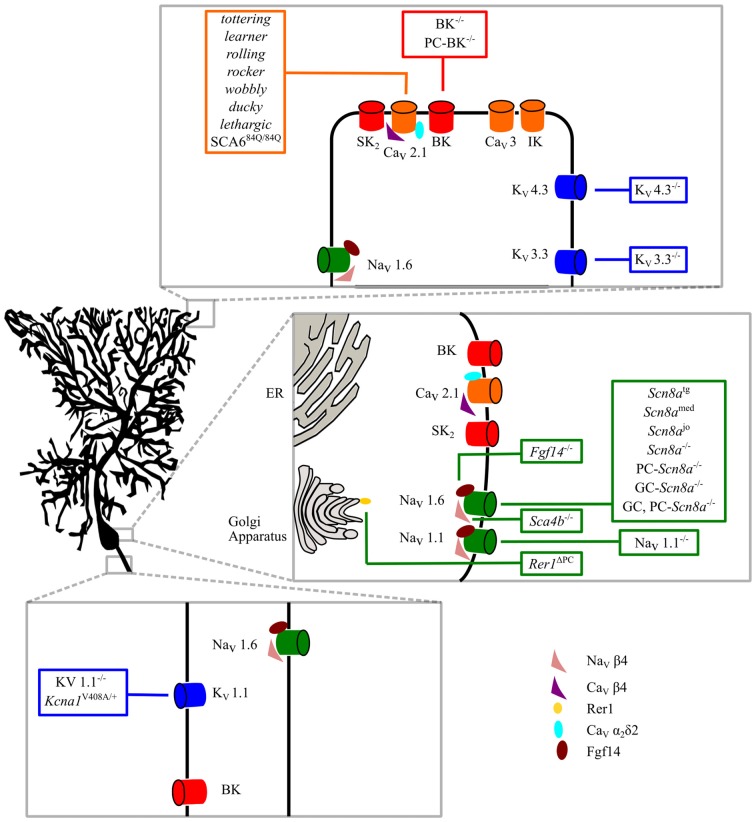
Intrinsic membrane mechanisms of Purkinje cell (PC) dysfunction. Ion channels involved in PC physiological functions are shown in the dendritic, somatic and axonal compartments. Ataxic mice with specific mutations are shown in the boxes. See the text for further explanation.

There is plenty of evidence that ion channel mutations affecting intrinsic membrane properties are responsible for numerous forms of ataxia (Figure 1), some of which have also been clinically described in humans. In most forms of ataxia, these mutations cause a reduced spontaneous firing of action potentials or lower excitability in PCs; whereas, in few cases, specific alterations of PC discharge variability or excessive excitability have been reported (Hoxha et al., 2013). However, the type and the extent of firing alterations necessary to cause motor symptoms have never been systematically investigated. Moreover, in the majority of cases, the mutation triggering ataxia does not reproduce the entire spectrum of motor symptoms, resulting in a variety of clinical presentations. In addition, nearly all studies only assessed a few motor parameters, so that at present it is not possible to define a relationship between a specific aspect of PC dysfunction and single features of motor deficits. The uncertainty is increased by the fact that in several animal models of ataxia, different laboratories have found different patterns of alteration, i.e., uniform reduction of PC firing rate vs. preserved excitability in some PCs but abolishment of firing in others.

**Figure 2:**
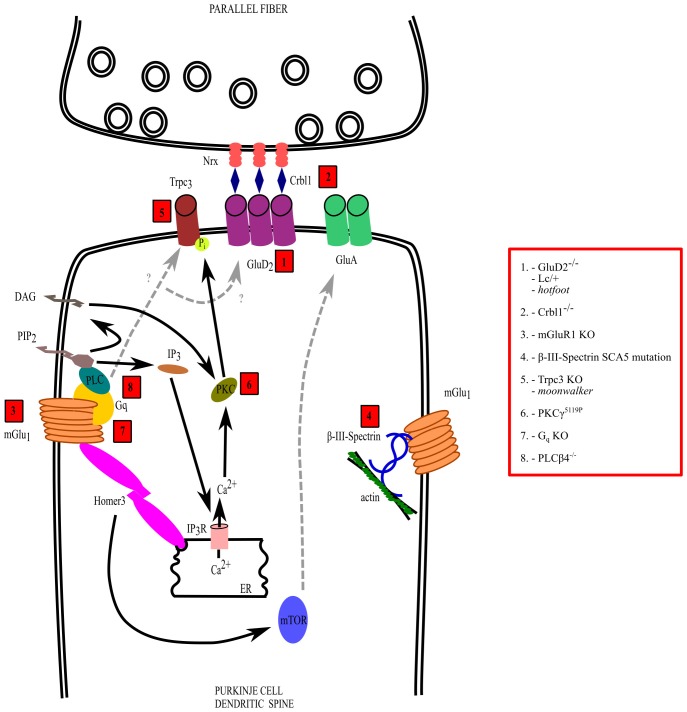
Mechanisms of dysfunction of the parallel fiber (PF)-PC synapse. The interactions and transduction pathways of glutamate delta-2 (GluD2) and metabotropic glutamate receptor 1 (mGlu1) receptors are shown in a PC dendritic spine postsynaptic to a PF synaptic varicosity. Numbers in red boxes refer to the molecules that are mutated or deleted in ataxic mice, which are listed in the red box on the right side, outside the drawing. See the text for further explanation.

In addition to intrinsic membrane properties, several mutations affecting the development and/or function of synaptic circuits, particularly of PF and/or CF to PC synapses, have been revealed in hereditary and acquired forms of ataxia (Figure 2). Such mutations can involve several receptors expressed on PCs such as the metabotropic glutamate receptor 1 (mGlu1) and the delta 2 receptor (glutamate delta-2, GluD2), causing motor deficits with different severity and with specific features, depending of the transduction pathway involved or on the type of postsynaptic signaling alteration. In addition, ataxia can be due to disruption of molecules involved in the CF synapse refinement and maturation, such as semaphorins or BDNF.

## Ataxia Due to Alterations of Purkinje Cell Intrinsic Membrane Properties

### Physiological Mechanisms of Purkinje Cell Firing

PCs *in vivo* spontaneously fire action potentials also in resting conditions and under anesthesia (Granit and Phillips, 1956; Bell and Grimm, 1969; Savio and Tempia, 1985). PC action potentials are of two types: simple spikes, which are similar to those fired by other types of neuron; and complex spikes, consisting of an initial spike immediately followed by a series of small spike oscillations superimposed on a sustained depolarization (Eccles et al., 1966). Complex spikes are generated by the activity of the CF–PC synapse, whereas simple spikes are driven by PF input.

Periods of firing are periodically interrupted by epochs in which PCs are silent and hyperpolarized, due to bistability of their membrane potential (Williams et al., 2002; Loewenstein et al., 2005; Engbers et al., 2013b). Bistability refers to the property of having two distinct values at which the membrane potential is stable. In the case of PCs, at the more depolarized potential they generate tonic simple spike firing, while at the more hyperpolarized membrane potential they are silent. Indeed, PC firing is mainly determined by the intrinsic membrane properties and does not require synaptic activation to occur (Jaeger and Bower, 1999). PCs spontaneously generate action potentials even when excitatory synapses are blocked in slice preparations (Häusser and Clark, 1997), in culture (Gruol and Franklin, 1987) and after acute isolation of the cell body (Nam and Hockberger, 1997). Such a pacemaking activity may determine a very constant and regular firing. However, PC spontaneous firing *in vivo* is highly irregular, because it is shaped by incoming signals coming from PF and CF synapses (Granit and Phillips, 1956) as well as by inputs from GABAergic interneurons (Häusser and Clark, 1997).

Another important feature of PC firing is its capability to attain high frequencies of discharge, a property that requires a special endowment of ion channels. The ionic mechanisms of PC firing, responsible for its peculiar functional features, have been investigated in detail. Spontaneous firing and high-frequency discharge are due to the interaction of voltage-dependent Na^+^ and K^+^ currents (Raman and Bean, 1999), while several other conductances modulate membrane excitability and shape the pattern of action potential firing.

### Purkinje Cell Sodium Channel Mutations

Voltage-gated sodium channels (Na_V_) are very important for setting the threshold for action potential initiation, axonal propagation of action potentials and integration of the synaptic inputs impinging on the cell. Ten genes encoding for α subunits (the pore-forming subunit) of Na_V_ channels have been described (Na_V_1.1–1.9; Goldin et al., 2000; Watanabe et al., 2000). They are expressed differently throughout the tissues and their expression is regulated during development. Among all the channels of the family, Na_V_1.1, Na_V_1.2, Na_V_1.3 and Na_V_1.6 are expressed exclusively in the central and peripheral nervous system (Goldin et al., 2000; Trimmer and Rhodes, 2004) with Na_V_1.3 having an embryonic expression. The Na_V_ channels expressed in the central nervous system differ for their subcellular localization; Na_V_1.1 and 1.3 have a somatic expression while Na_V_1.2 are localized in unmyelinated axons (Westenbroek et al., 1989, 1992). Na_V_1.6 channels are localized in myelinated axons and dendrites (Caldwell et al., 2000; Krzemien et al., 2000; Jenkins and Bennett, 2001).

In cerebellar PCs, Na^+^ currents are due to Na_V_1.1 and Na_V_1.6 channels (Westenbroek et al., 1989; Kalume et al., 2007). Na_V_1.1 channels are expressed in the cell body of PCs (Westenbroek et al., 1989), hence their importance for the control of membrane excitability, while Na_V_1.6 channels are mainly expressed in the soma, dendrites and initial segment of the axon (Westenbroek et al., 1989; Kalume et al., 2007). PC Na^+^ currents are tetrodotoxin (TTX) sensitive (Llinás and Sugimori, 1980), have a component of persistent current and during repolarization give rise to a resurgent current implied in pacemaker activity (Raman and Bean, 1997). In PCs, Na_V_1.1 channels contribute for about 60% of all components of Na^+^ currents, while Na_V_1.6 channels are responsible for the remaining part (Kalume et al., 2007). The resurgent Na^+^ current is mainly produced by the action of the auxiliary Na_V_β4 subunit on Na_V_1.1 and Na_V_1.6 channels (Grieco et al., 2005; Ransdell et al., 2017). The combined actions of the resurgent and persistent components of Na^+^ currents co-operate to confer spontaneous activity. It is interesting to note that the intracellular protein fibroblast growth factor 14 (FGF14), which controls Na_V_ channels’ membrane expression and localization by direct interaction with their C-terminal domain (Liu et al., 2001; Laezza et al., 2007), is required for PC spontaneous firing (Bosch et al., 2015). In contrast, the hyperpolarization-activated cationic current (I_H_), which in other types of neuron is essential for pacemaker activity, in PCs is not required for this function (Raman and Bean, 1999), but its role is to oppose the tendency of PCs to have a bistable membrane potential with periods of quiescence (Williams et al., 2002).

Mice with a complete deletion of Na_V_1.1 channels (Na_V_1.1 KO mice) revealed an ataxic phenotype with alterations of gait parameters in the footprinting test (both homozygous and heterozygous KO mice; Kalume et al., 2007). In PCs of both homozygous and heterozygous KO mice, the deletion of Na_V_1.1 caused a reduction of the persistent and resurgent sodium currents without changes in their biophysical properties. In line with the localization of Na_V_1.1 channels and the important role of persistent and resurgent Na^+^ currents in determining spontaneous firing, the authors reported a reduced spontaneous activity of PCs in Na_V_1.1 homozygous and heterozygous KO mice without changes in other action potential parameters (threshold, action potential amplitude). Furthermore, the injection of stronger depolarizing current was necessary to evoke action potential firing in Na_V_1.1 homozygous and heterozygous KO mice relative to their wild-type littermates (Kalume et al., 2007). Thus, the deletion of Na_V_1.1 channels caused a reduction of PC excitability that can, at least in part, explain the motor impairment observed in these mice.

In humans, mutations in the gene encoding the Na_V_1.6 channel (*SCN8A*), are associated with cerebellar atrophy and ataxia (Trudeau et al., 2006). Several mice with mutations in Na_V_1.6 have been described. Two *Scn8a* null mice have been considered as motor endplate disease models (Duchen and Searle, 1970; Duchen and Stefani, 1971; Burgess et al., 1995). The first one is the *Scn8a*^med^ mouse, in which the deletion is due to a spontaneous insertion of an L1 element in the exon 2 of the *Scn8a* gene (Kohrman et al., 1996a). The second one is the *Scn8a*^tg^ mouse, generated by a nontargeted transgene insertion in the *Scn8a* gene (Kohrman et al., 1995). Among all observed deficits, both these mutant mice presented with ataxia (Duchen and Stefani, 1971). Another Na_V_1.6 mutant, the *jolting* mouse (*Scn8a*^jo^) carrying a missense mutation (Kohrman et al., 1996b), was described in 1965 with uncoordinated gait (Dickie, 1965). Isolated PCs from *Scn8a*^med^ and *Scn8a*^jo^ mice, showed a reduced amplitude of the steady-state and resurgent components of sodium currents (Raman et al., 1997). PCs from *Scn8a*^med^ and *Scn8a*^jo^ mice showed reduced spontaneous firing (Harris et al., 1992; Raman et al., 1997), an observation which is in line with the fact that the resurgent Na^+^ current is very important in sustaining spontaneous and high-frequency firing (Raman et al., 1997). In the global null mice for Na_V_1.6, the severe phenotype observed was due to functional deficits of different regions of the nervous system, so that it is difficult to ascribe motor impairment to cerebellar dysfunctions only. Very important in defining the link between altered PC firing and ataxia was the generation of conditional mutant mice lacking Na_V_1.6 channels exclusively in PCs (PC KO) and/or granule cells (GC KO and double KO; Levin et al., 2006). PC KO mice developed mild ataxia with impairment in the rotarod test and alterations of the gait. Double KO mice developed a severe ataxia with compromised gait. PCs from both PC KO and double KO mice were characterized by a reduction of the resurgent Na^+^ current reflected in a reduced evoked action potential firing (Levin et al., 2006). These reports are in line with previous data on global null mice for Na_V_1.6 obtained from isolated PCs (Harris et al., 1992; Raman et al., 1997). In contrast to the ataxic signs observed in PC KO and double KO mice, GC KO mice did not display any ataxic sign. Furthermore, the resurgent current and the firing frequency of PCs of GC KO mice were comparable to those of wild-type PCs (Levin et al., 2006).

Interestingly, more evidence comes from studies in mice that do not have specific mutations in Na_V_ channels, but in auxiliary subunits like Na_V_β4 (Ransdell et al., 2017) and Fgf14 (Shakkottai et al., 2009; Bosch et al., 2015), or in the sorting receptor Rer1 (*Rer1*^Δ*PC*^; Valkova et al., 2017). Mice with a selective PC deletion of the Na_V_β4 subunit showed deficits in motor coordination and balance associated with a marked reduction of the Na^+^ resurgent current and spontaneous and evoked firing impairment (Ransdell et al., 2017). These results demonstrate that, in PCs, Na_V_β4 is essential for the resurgent current and for action potential repetitive firing. Mice with deletion (Shakkottai et al., 2009) or knockdown (Bosch et al., 2015) of *Fgf14* showed an ataxic phenotype, a reduced number of Na_V_1.6 channels and a deficit in spontaneous firing. Rer1 is a sorting receptor of the cis-Golgi, very important for the quality control of the complexes transported in the membrane (Füllekrug et al., 1997; Valkova et al., 2011). The specific deletion of *Rer1* in PCs led to an ataxic phenotype (Valkova et al., 2017). This was accompanied by defects in high-frequency firing and a reduction in the expression of Na_V_1.1 and Na_V_1.6 channels.

These data indicate that a specific disruption of PC firing is sufficient to cause an ataxic phenotype whether this disruption is due to a direct mutation of ion channels or to an indirect action on their activity or expression. However, the study of ataxia in models with mutated Nav channels has been complicated by the expression of these channels in many other regions of the central nervous system, where their deletion has additional consequences. In fact, Nav1.1 loss of function mutations cause ataxia associated with severe infantile myoclonic epilepsy (Sugawara et al., 2002) and Nav1.1 KO mice reproduce both clinical features (Kalume et al., 2007). Mutations of Nav1.6, in addition to ataxia, cause mental retardation and attention deficit disorder (Trudeau et al., 2006). Animal models with gene deletion selective for cerebellar PCs (Nav1.6, Levin et al., 2006; Fgf14, Bosch et al., 2015; Na_V_β4, Ransdell et al., 2017) have been very useful to clarify which deficits were specifically due to PC dysfunction. It is interesting to note that, in the case of Nav1.6, PC selective deletion caused only a mild ataxia, while GC selective deletion produced no symptoms, but the double KO showed severe ataxia (Levin et al., 2006), suggesting an additive effect of Nav1.6 ablation in GCs. In PCs, the loss or dysfunction of Nav channels has the peculiar feature of reducing the Na^+^ resurgent current, with a consequent impairment of firing. For this reason, ataxia due to a primary defect of Na^+^ currents might be considered as a special category, in which the main determinant is a loss or attenuation of the resurgent current.

### Purkinje Cell Potassium Channel Mutations

#### High Threshold Potassium Channels

In order to attain high firing frequencies, Na^+^ currents must interact with K^+^ conductances endowed with fast activation and deactivation properties. The high-voltage-activated potassium channels of the Kv3 subfamily possess these properties (Rudy and McBain, 2001). The four Kv3 subunits are widely expressed in the brain (Weiser et al., 1994). PCs express three members of this family, Kv3.1, Kv3.3 and Kv3.4 (Martina et al., 2003; Sacco et al., 2006), among which the most highly expressed is Kv3.3 (Weiser et al., 1994; Boda et al., 2012). In the cerebellar cortex, Kv3.3 is specific of PCs and it is expressed at the same age at which they acquire their fast-spiking phenotype (Goldman-Wohl et al., 1994; Boda et al., 2012). Moreover, the deletion of the Kv3.3 gene (*KCNC3*) disrupts PC high frequency firing, which is rescued by its re-introduction by the dynamic clamp technique (Akemann and Knöpfel, 2006). Thus, Kv3 K^+^ currents associated with the resurgent Na^+^ current provide a mechanistic explanation of high-frequency firing in PCs (Akemann and Knöpfel, 2006).

Kv3.3 KO mice show mild deficits in walking trajectories with an increased lateral deviation while ambulating. Furthermore, as an indication of motor impairment, Kv3.3 KO mice show an increased number of slips in the balance beam test (Joho et al., 2006). These ataxic signs are in line with the electrophysiological alterations found in the cerebellum of Kv3.3 KO mice. McMahon et al. (2004) reported that action potentials were broader in Kv3.3 KO than in wild-type PCs. Furthermore, the action potentials of Kv3.3 KO PCs present with large amplitude and reduced fast afterhyperpolarization (AHP). In PCs of Kv3.3 KO mice, the increased duration of action potentials is accompanied by increased interspike intervals, thus a reduced spontaneous frequency, and the inability to sustain high frequency firing (Hurlock et al., 2008). PCs of Kv3.3 KO mice also display a reduced number of spikelets in complex spikes induced by CF stimulation. The importance of the Kv3.3 channel for PC proper function in order to have a normal motor performance is highlighted by the experiments conducted by Hurlock et al. (2008) who restored Kv3.3 expression exclusively in PCs. This specific expression completely reverted electrophysiological alterations observed in Kv3.3 KO PCs. Moreover, the lateral deviation and motor deficits in the balance beam test were abolished in rescued animals.

Missense mutations in the *KCNC3* gene that encodes the Kv3.3 channel in humans are associated with autosomal dominant spinocerebellar ataxia type 13 (SCA13; Waters et al., 2006; Figueroa et al., 2010, 2011). Expression of human Kv3.3 mutations in murine cerebellar cultures caused a reduction of the total outward K^+^ current, a broadened action potential and a reduction of PC excitability (Irie et al., 2014).

Overall, these studies demonstrate the importance of normal PC functioning and how subtle action potential alterations are sufficient to cause ataxia. In the specific case of the Kv3 current, impairment PC firing dysfunction is due to a slower repolarization of action potentials and to the loss of the AHP. The combined action of these two deficits hampers high frequency firing, limiting the range of PC output signals sent to the deep cerebellar nuclei. The interpretation of this finding is complicated by the fact that also spontaneous PC firing is reduced in Kv3.3 KO mice (Hurlock et al., 2008). It is noteworthy that, in spite of the combination of deficits in spontaneous PC firing, high frequency discharge, complex spike waveform and deep cerebellar nuclei firing impairment, the motor deficits are quite mild and only detectable with specific and sensitive tests.

#### Subthreshold Potassium Channels

Subthreshold, inactivating K^+^ currents of PCs are generated by Kv1, Kv4 and Kv11 channels (Sacco and Tempia, 2002; Sacco et al., 2003). In PCs, dendritic Kv1 channels prevent the generation of random spontaneous Ca^2+^ spikes (Khavandgar et al., 2005). Kv4 channels located in the dendrites filter high frequency incoming synaptic signals (Hoffman et al., 1997). In PCs, the Kv4.3 channel is located in the soma and in the dendrites (Wang and Schreurs, 2006). Potassium channels of the Kv11 subfamily (also known as Erg) are highly expressed in PCs (Guasti et al., 2005), where they modulate membrane excitability and firing frequency adaptation (Sacco et al., 2003).

The Kv1 voltage-gated potassium channel family includes eight α subunits (Kv1.1–Kv1.8; Coetzee et al., 1999; Hille, 2001; Yu and Catterall, 2004). In the brain, the Kv1 subunits are localized to the axon initial segment, juxtaparanodes and synaptic terminals (Trimmer, 2015), where they exert a crucial role in the axonal membrane repolarization after an action potential, in adjusting the resting membrane potential and in controlling neurotransmitter release (Hille, 2001; Jan and Jan, 2012). Kv1 subunits can assemble together to form hetero-tetramers that confer to Kv1 channels a great diversity of functional properties (Ovsepian et al., 2016). In the cerebellum, the low threshold delayed rectifier subunits Kv1.1 and Kv1.2 are expressed in the terminals of basket cells (McNamara et al., 1993; Wang et al., 1993, 1994; Laube et al., 1996; Chung et al., 2001) and at least Kv1.2 in the dendrites of PCs (Khavandgar et al., 2005).

Kv1.1 KO mice display a reduced ability to maintain balance on a stationary rod (Smart et al., 1998; Zhang et al., 1999). Zhang et al. (1999) showed in Kv1.1 KO mice an increased frequency of spontaneous inhibitory postsynaptic currents (sIPSCs) recorded from PCs, causing an excessive tonic inhibition of PCs. Several point mutations of the *KCNA1* gene coding for the Kv1.1 subunit (Browne et al., 1994; reviewed in Ovsepian et al., 2016), have been reported in patients suffering from episodic ataxia type 1 (EA1), an autosomal dominant neurological disease with paroxysmal cerebellar ataxia, epilepsy, myokymia (Browne et al., 1994). A heterozygous knock-in mouse model for EA1 (*Kcna1*^V408A/+^) was created by Herson et al. (2003). The authors reported a reduction in frequency and amplitude of sIPSCs recorded from PCs of *Kcna1*^V408A/+^ mice and stress-induced motor impairment (Herson et al., 2003). In both KO and knock-in mice the authors failed to find a change in the firing frequency of basket cells (Zhang et al., 1999; Herson et al., 2003), despite the Kv1.1 localization to perisomatic baskets of PCs (Wang et al., 1993, 1994; Laube et al., 1996; Chung et al., 2001). However, an increase in the action potential width was found in presynaptic boutons of basket cells in *Kcna1*^V408A/+^ mice (Begum et al., 2016). The paired pulse ratio of evoked IPSCs of PCs was lower in *Kcna1*^V408A/+^ mice, indicating an increased release probability (Begum et al., 2016). Such an increased inhibitory tone was accompanied by a reduction in spontaneous firing activity of PCs (Begum et al., 2016).

A missense mutation of Kv1.2, which is often assembled with Kv1.1, causes ataxia in mice (Xie et al., 2010). PCs of Kv1.2 mutant mice (named *Pingu* or *Pgu*) showed an increased frequency and amplitude of inhibitory postsynaptic potentials (IPSPs), causing a reduced PC firing frequency. Mutations of Kv1 channels, in addition to effects on the axon terminals of basket cells, might also directly alter the dendritic excitability of PCs. In fact, application of a selective Kv1 blocker causes random transient increases of PC firing, driven by an uncontrolled action of dendritic Ca^2+^ spikes (Khavandgar et al., 2005).

The major component of the subthreshold inactivating K^+^ current in PCs is due to Kv4 channels (Sacco and Tempia, 2002; Hourez et al., 2011). The only Kv4 subunit expressed by PCs is Kv4.3 (Serôdio and Rudy, 1998). The Kv4.3 channel is localized to PC dendrites, where it is associated with Ca_V_3 Ca^2+^ channels (Anderson et al., 2013). Mutations of *KCND3*, the gene encoding the Kv4.3 subunit, cause the SCA19 (Duarri et al., 2012, 2015; Lee et al., 2012). A more complex syndrome, in which early onset ataxia is associated with intellectual disability, epilepsy, attention deficit hyperactivity disorder, strabismus, oral apraxia and joint hyperlaxity is caused by a mutation that shifts the Kv4.3 activation curve to very depolarized potentials (Smets et al., 2015). Although a Kv4.3 KO mouse has been created (Niwa et al., 2008), no motor or neurologic test was performed.

The pathophysiological mechanisms resulting from subthreshold K^+^ channels mutations are quite heterogeneous. In fact, Kv1 channels mutations mainly affect the GABAergic tone exerted by inhibitory interneurons on PCs, decreasing their activity. The ataxia resulting from Kv1 mutations has an episodic nature, likely due to such peculiar cellular mechanism. In contrast, Kv4.3 mutations have pleiotropic effects on SCA19/22 patients, encompassing a continuous early onset ataxia, intellectual disability, epilepsy, attention deficit hyperactivity disorder and other features. Since an animal model of SCA19/22 is not yet available, it is not possible to assign these symptoms to a specific alteration of PC excitability.

#### Calcium- and Voltage-Dependent BK Potassium Channels

Large conductance voltage- and Ca^2+^-activated K^+^ channels (also called Big K^+^ channels or BK channels) have a double gating mechanism. In fact, the voltage-dependance curve of their gating is shifted to negative potentials by an increase in intracellular Ca^2+^ concentration, allowing them to open whenever a depolarization is associated with Ca^2+^ entry (Hille, 2001). These channels introduce a brief hyperpolarization period between action potentials, necessary to prevent the cell to reach the firing threshold too early. BK channels are expressed throughout the brain especially in excitable cells (Knaus et al., 1996; Sausbier et al., 2006).

In PCs (Gähwiler and Llano, 1989; Gruol et al., 1991; Knaus et al., 1996) and in cerebellar Golgi cells (Sausbier et al., 2006), BK channels are localized to the soma and dendrites. In PCs, BK K^+^ channels give rise to two types of current: fast-gated, inactivating and slow-gated, non-inactivating (Benton et al., 2013). The former type activates on the time scale of an action potential so that it contributes to spike repolarization (Edgerton and Reinhart, 2003). In contrast, slow-gated, non-inactivating BK currents open during the AHP and contribute to a sustained interspike conductance shaping the PC firing pattern (Womack et al., 2009; Benton et al., 2013). In addition to such somatodendritic functions, in PCs BK channels are localized to the paranodal regions of Ranvier nodes, where they are essential to allow high fidelity propagation of action potentials (Hirono et al., 2015).

New insights for the importance of BK channels in the cerebellum come from the studies of Sausbier et al. (2004), who performed a deletion of the pore exon of the α subunit of BK channels thus generating mice lacking functional BK channels (BK^−/−^ mice). BK^−/−^ mice exhibited abnormal gait with shorter stride length and irregular step pattern in the footprinting test (Sausbier et al., 2004). In the beam balance test, BK^−/−^ mice crossed the beam with a greater number of foot slips than their wild-type littermates. Furthermore, when tested in the rotarod test, BK^−/−^ mice showed a reduced latency to fall off the rod relative to their wild-type littermates. At the cerebellar-specific eye-blink conditioning test, BK^−/−^ mice showed no learning. Sausbier et al. (2004); with current clamp recordings, found a reduced AHP in PCs from BK^−/−^ mice. Furthermore, the majority of PCs from BK^−/−^ mice lacked spontaneous discharge and the overall evoked action potential frequency was reduced. The authors reported that, in BK^−/−^ mice, PCs were silent because of a depolarized resting membrane potential responsible for the inactivation of Na^+^ channels. These findings suggest an important role of PC BK channels for normal cerebellar function. However, BK channels are also expressed in other cells of the cerebellum (Knaus et al., 1996), hence it is difficult to determine if the ataxic phenotype is directly linked to PC firing alterations. Chen et al. (2010) developed a murine model, which lacked BK channels in PCs (PC-BK^−/−^). PC-BK^−/−^ mice displayed motor alterations similar to those observed in global BK^−/−^ mice although with a reduced severity (Sausbier et al., 2004). PC-BK^−/−^ mice showed gait alterations on footprinting test, disrupted motor coordination and balance on ladder runway and balance beam tests (Chen et al., 2010). *In vivo* recordings highlighted a slight reduction in the simple spike frequency of PCs and a strong reduction of complex spikes. The reduced activity of PCs determined an increased activity in deep cerebellar nuclei, which might be the cause of the reduced complex spike generation.

The mechanism of ataxia due to the lack of BK channels differs from the previously described mutations, because it is based on an excessively depolarized resting potential causing Na^+^ channel inactivation and block of action potential generation. Thus, mutations in the superfamily of K^+^-selective channels, by hampering PC firing in a different manner, result in ataxia, but with different and specific features, ranging from the episodic nature in Kv1 mutants to an extensive syndrome in Kv4 loss of function to a more classical form of ataxia following BK deletion.

### Purkinje Cell Calcium Channel Mutations

Voltage-gated calcium channels (Ca_V_ channels) are responsible for Ca^2+^ entry in the cell in response to membrane depolarization. There are different subclasses of Ca_V_ channels, which exert important roles in different cell types (Hille, 2001). In the central nervous system, Ca_V_ channels control several processes including presynaptic neurotransmitter release, neuritogenesis and gene expression (Kamp et al., 2012). Among Ca_V_ channels, the P/Q-type (Ca_V_2.1) is widely expressed in the brain (Westenbroek et al., 1995; Craig et al., 1998).

Ca^2+^ channels of PCs are highly enriched in dendrites, as shown by Ca^2+^ imaging (Ross and Werman, 1987; Tank et al., 1988) and immunohistochemistry (Westenbroek et al., 1995; Yokoyama et al., 1995; Indriati et al., 2013) studies. The most represented Ca^2+^ current is Ca_V_2.1 (P/Q type), accounting for more than 90% of Ca^2+^ currents, with the remaining fraction due to Ca_V_1 (L type) channels (Regan, 1991; Usowicz et al., 1992). In PCs, clusters of Ca_V_2.1 channels are co-localized with voltage/Ca^2+^-dependent BK channels and type 2 small conductance calcium-dependent K^+^ channels (SK2; Womack et al., 2004; Indriati et al., 2013), constituting nanodomains of interaction between Ca^2+^ entry and the activation of K^+^ conductances. Ca_V_3 channels, responsible for T-type Ca^2+^ currents, are located in PC dendrites, where they give rise to Ca^2+^ signals upon activation of the PF-PC synapse (Ly et al., 2016). Moreover, in PCs Ca_V_3 channels are associated with intermediate conductance Ca^2+^-dependent K^+^ channels (IKCa; Engbers et al., 2013a). The Ca_V_3/IK_Ca_ complex allows subthreshold activation of an outward K^+^ current, which exerts a strong dampening of temporal summation of PF-excitatory post-synaptic potentials (EPSPs), so that only the first few events undergo summation with a marked suppression of subsequent summation (Engbers et al., 2013a). This mechanism allows a selective transmission of brief bursts of PF input signals.

Mutations of the α subunit of the Ca_V_2.1 channel, in humans, are linked to familial hemiplegic migraine-1, episodic ataxia type 2 (EA2), and to spinocerebellar ataxia type 6 (SCA6; Ophoff et al., 1996; Zhuchenko et al., 1997; Pietrobon, 2010). Spontaneous mutations of the Ca_V_2.1 channel, in mice, gave rise during years to several animal models to study the EA2 disease: mice with point mutations in the α1A subunit of the Ca_V_2.1 channel, *tottering* (Fletcher et al., 1996), *leaner* (Tsuji and Meier, 1971), *rolling* (Oda, 1973), *rocker* (Zwingman et al., 2001), and *wobbly* (Xie et al., 2007); mice with mutations in the ancillary β4 subunit (*lethargic*, Burgess et al., 1997) and mice with mutations in the α2δ2 subunit (*ducky*, Barclay et al., 2001). In PCs of *ducky*, *leaner* and *tottering* mice, these mutations caused a reduced calcium current, decreasing the firing frequency and the precision in pacemaking activity, which might be the cause of the ataxic phenotype (Donato et al., 2006; Walter et al., 2006). Similar results were obtained also by Watase et al. (2008) that generated a knock-in mouse model, to study the SCA6 disease, by the insertion of 84 CAG human repeats in the murine locus of the Cacna1a gene (SCA6^84Q^). SCA6^84Q^ mice developed motor coordination deficits in adult age (Watase et al., 2008; Jayabal et al., 2015). Extracellular recordings of cerebellar slices from both homozygous SCA6^84Q/84Q^ and heterozygous SCA6^84Q^ mice revealed a reduction of the precision of the action potential timing of the PC. Furthermore, SCA6^84Q/84Q^ mice also showed a reduced range of PC firing frequency (<100 Hz; Jayabal et al., 2016). The colocalization of Ca_V_2.1 channels with Ca^2+^-dependent K^+^ channels might explain the reduced precision in the pacemaking activity of PCs (Womack et al., 2004). The reduced Ca^2+^ entry in the cell observed in these different mouse models probably leads to a reduced activation of Ca^2+^-dependent K^+^ channels, and as a consequence, the cell acquires a depolarized membrane potential that blocks the Na^+^ channel activation (Donato et al., 2006). Indeed, a reduced spontaneous activity due to a depolarized membrane potential was observed also in BK^−/−^ mice (Sausbier et al., 2004). The important role of Ca^2+^-dependent K^+^ channels in the precision of pacemaking activity of PCs in Ca_V_2.1 mutant mice was further investigated by Walter et al. (2006). They demonstrated that bath perfusion of cerebellar slices from *ducky* mice with a SK channel activator was able to reduce the coefficient of variation of the firing rate and to increase the AHP amplitude to the size necessary for the recovery of Na^+^ channels from inactivation. Furthermore, *in vivo* treatments of *ducky* and *tottering* mice with the SK channel activator resulted in an improvement of their motor performance (Walter et al., 2006).

Cav channel mutations in PCs can result in hemiplegic migraine or ataxia. The latter can be either episodic when due to missense mutation (EA2), or classic ataxia following introduction of CAG repeats (SCA6). The different functional alteration of the channel might be responsible for the specific phenotype of the disease. It is interesting to note that ataxia due to Cav2.1 mutation causing reduced Ca^2+^ entry, leads to insufficient activation of BK channels. In fact, the mechanism is very similar to a direct loss of function of BK. The peculiar feature of these ataxias is the loss of precision in PC firing. However, it is far from clear why variability in PC firing must remain within a narrow range and how changes in its firing precision affect output signals from the cerebellar cortex during movement.

## Ataxia Due to Alterations of the Parallel-Fiber/Purkinje Cell Synapse

### Alterations of the Purkinje Cell GluD2 Receptor Pathway

In the cerebellum, the delta-2 receptor (GluD2) is expressed post-synaptically on PCs at the PF-PC synapse (Takayama et al., 1996; Landsend et al., 1997). In terms of protein sequence analogy, the GluD2 receptor belongs to the ionotropic glutamate receptor family (Yamazaki et al., 1992), but it is not activated by glutamate (Hirai et al., 2005). Matsuda et al. (2010) have identified cerebellin 1 (Cbl1), a protein secreted from granule cells, as a ligand of the GluD2 receptor.

Mutations in the *GRID2* gene encoding for GluD2 have been recently associated with cerebellar ataxia in several patients (Hills et al., 2013; Utine et al., 2013; Maier et al., 2014; Coutelier et al., 2015; Van Schil et al., 2015; Ali et al., 2017). The link between GluD2 and ataxia is strongly supported by several studies in mice with different mutations in the *Grid2* gene, that have provided important information about the role of the GluD2 receptor in cerebellar functions and how their mutations affect cerebellar circuitry and cause ataxia. Spontaneous mutations include the *lurcher* (Lc/+) semi-dominant point mutation (Zuo et al., 1997) and the *hotfoot* (*ho*) autosomal recessive mutations (described below; Lalouette et al., 1998; Wang et al., 2003; Miyoshi et al., 2014). In addition, mice with a specific gene-targeted deletion of *Grid2* have been created (GluD2^−/−^; Kashiwabuchi et al., 1995).

The Lc mutation leads to the death of PCs and to the loss of the majority of granule cells in Lc/+ mice (Zuo et al., 1997). Lc/+ PCs have a depolarized resting membrane potential due to a large constitutive inward current (Zuo et al., 1997) that might be the cause of such massive neuronal death in the cerebellum. On the contrary, no cerebellar PC or granule cell degeneration is present on GluD2^−/−^ or *ho* mice, which display a similar pattern of cerebellar alterations, consistent with loss of function mutations (Guastavino et al., 1990; Kashiwabuchi et al., 1995; Motohashi et al., 2007).

Detailed studies on GluD2^−/−^ mice have demonstrated that GluD2 plays as in important role in the stabilization of the PF-PC synapse, restriction of the CF innervation to the proximal dendritic domain of the PC and regulation of long-term depression (LTD). In fact, GluD2^−/−^ mice have an impaired PF synaptogenesis, with a reduction in the number of spines between PF-PC, and an increase of free spines (Kashiwabuchi et al., 1995; Kurihara et al., 1997). Furthermore, the presence of free spines was observed also in GluD2^ho4J/ho4J^ (Lalouette et al., 2001). The alterations of the PF-PC synapse are in accordance with the demonstration that the GluD2 receptor is part of the protein complex neurexin-Crbl1-GluD2, critically involved in the formation and maintenance of PF-PC synapses *in vivo* (Ito-Ishida et al., 2008; Kakegawa et al., 2009). The extracellular N-terminal domain of the GluD2 receptor expressed in PCs binds the presynaptic protein neurexin through Crbl1 secreted from PFs, creating a bridge that acts as a bidirectional synaptic organizer at the PF-PC synapse (Matsuda et al., 2010; Uemura et al., 2010). Any alteration of the proteins forming the trio led to the disruption of the PF-PC synapse. Indeed, it has been reported that Crbl1 null mice have a reduced number of PF-PC synapses and an increase of free spines (Hirai et al., 2005), a phenotype similar to that of GluD2^−/−^ mice.

GluD2^−/−^ mice have a deficit in the elimination of surplus CFs during development (Kashiwabuchi et al., 1995; Kurihara et al., 1997). Ichikawa et al. (2002) showed that CFs of GluD2^−/−^ mice extended into the distal domain of the PC dendritic tree, invading spiny branchlets, which are normally innervated by PFs only. The authors suggest that the reduction of the PF-PC synapses in the distal part of the dendritic tree of PCs and the appearance of free spines permit the extension of the CF into the PF territory. These findings suggest an important role of GluD2 for the restriction of the CF innervation to the proximal dendritic domain of the PC (Ichikawa et al., 2002).

The LTD impairment observed in GluD2^−/−^ mice is in line with other studies, in which the gene was knocked down Jeromin et al. (1996) and with *in vitro* studies on cerebellar cultures, where the GluD2 receptor was silenced with antisense nucleotides (Hirano et al., 1994). The use of an antibody against the H2 domain (putative ligand-binding domain) of the GluD2 receptor on cultured PCs decreased the clusters of synaptic AMPA receptors and the amplitude of excitatory post-synaptic currents (EPSCs; Hirai et al., 2003). Moreover, treatment of PCs with the H2 antibody completely blocked LTD in wild-type PCs (Hirai et al., 2003). Genetic reintroduction of GluD2 in the GluD2-deficient PC rescued LTD induction (Hirai et al., 2005; Yawata et al., 2006). These data confirm the crucial role of GluD2 in the induction of LTD even if the exact mechanism is not yet known.

The more debated role of the GluD2 receptor is its function as ion channel and its gating mechanism. The presence of a large sustained constitutive inward current in Lc/+ PCs suggested that this mutation blocked the putative GluD2 channel in an open conformation (Zuo et al., 1997; Kohda et al., 2000; Schmid et al., 2009). Kato et al. (2012) demonstrated that the GluD2 receptor associates with mGlu1 and TRPC3 and regulates mGlu1-mediated synaptic transmission. More recently, Ady et al. (2014) provided evidence that activation of the mGlu1 receptor triggers the opening of the GluD2 channel.

### Alterations of the Purkinje Cell mGlu1 Receptor Pathway

Studies in humans and mice have demonstrated that dysfunctions of mGluR1 and its downstream signaling cascade are implicated in the pathogenesis of hereditary and acquired forms of ataxia. The mGlu1 receptor, encoded by *GRM1*, is a G protein-coupled glutamate receptor, highly expressed in PCs outside the postsynaptic density and activated by high repetitive stimulation of PFs (Batchelor et al., 1994; Batchelor and Garthwaite, 1997; Tempia et al., 1998). mGluRs play a critical role in LTD induction and motor learning as well as the elimination of redundant CF synapses, that takes place during the first three postnatal weeks in mice (Conquet et al., 1994; Kano et al., 1997, 2008; Hoxha et al., 2016). Activation of mGluR1 generates slow EPSPs/EPSCs mostly mediated by the non-selective cation-permeable transient receptor potential channel TRPC3 (Hartmann et al., 2008). In addition, mGlu1 receptor activation determines local Ca^2+^ transients in PC dendrites via a signaling cascade involving Gq/11 protein, phospholipase C, diacylglycerol and inositol 1,4,5-trisphosphate (IP3); IP3 induces Ca^2+^ release from intracellular stores (Finch and Augustine, 1998; Takechi et al., 1998; Tempia et al., 2001).

Sequencing analysis carried out in families affected by forms of congenital cerebellar ataxia have identified splicing mutations of the *GRM1* gene, which result in aberrant transcripts encoding nonfunctional mGlu1 (Guergueltcheva et al., 2012; Watson et al., 2017). In addition, antibodies against mGlu1 have been identified in patients affected by cerebellar ataxia caused by tumors or metastases (Sillevis Smitt et al., 2000). Interestingly, the injection of purified IgG from serum and cerebrospinal fluid of patients with paraneoplastic cerebellar ataxia into the subarachnoid space of normal mice caused ataxia (Sillevis Smitt et al., 2000). The ataxic behavior was evident in mice 3 h after injection of IgG and persisted for 24 h.

A direct link between ataxia and mGlu1 receptors comes from studies on mGluR1 KO mice. These mice develop ataxic gait together with impaired cerebellar CF synapse elimination and deficient LTD; all these phenotypes can be rescued by introducing the mGluR1 transgene specifically in the PCs (Kano et al., 1997; Ichise et al., 2000).

On the other hand, mGluR1* loss of function* has been unveiled in a large number of animal models of human cerebellar ataxia such as SCA1 transgenic mice carrying an expanded number of CAG repeats in the ataxin gene, which results in a poly-glutamine tract (polyQ) expansion in the ataxin-1 protein (Serra et al., 2004; Orr, 2012). Such mutation prevents ataxin-1 interaction with the retinoid-related orphan nuclear receptor-alpha (RORα) resulting in a decrease in RORα-mediated transcriptional activity and eventually downregulation of mGluR-related genes (Serra et al., 2006). SCA1^*154Q*^ mice, with 154 CAG repeats in the ataxin-1 gene, show reduced expression of mGluR1 on PC dendrites associated with an abnormal increase of mGluR5 expression, which is normally undetectable in the cerebellum of adult animals (Watase et al., 2002; Notartomaso et al., 2013). Since mGlu5 receptors are highly expressed in the early postnatal life, their persistent expression in the adulthood may be indicative of an immature cerebellum state (Casabona et al., 1997).

Recent studies in SCA1^154Q^ mice have demonstrated a significant reduction of Homer-3 expression in PC dendrites and spines at the early age, while mGluR1 expression levels are still unaltered (Ruegsegger et al., 2016). Interestingly, Homer 3 is a scaffold protein localized in the postsynaptic density involved in the coupling of mGluR1 and IP3 receptors (IP3Rs) responsible for the Ca^2+^ release from endoplasmic reticulum (ER) stores in PCs (Tu et al., 1998; Willard and Koochekpour, 2013). Homer 3 is also implicated in the mTORC1 signaling pathway activation and translation of synaptic proteins including Homer 3, thus regulating synaptic plasticity (Ruegsegger et al., 2016). The deletion of mTORC1 in SCA1^154Q^ reduced Homer-3 levels and exacerbated pathology, anticipating the motor coordination impairment. Rescuing Homer-3 levels in PCs improved motor deficits, enhanced mTORC1 signaling in PCs, and reduced the spine loss.

In the ataxic SCA1^82Q^ mouse model, with PC-specific 82Q repeats in the ataxin-1 gene, the progressive decline of mGluR1 function begins at the early stage of disease (at 5 weeks of age) before the onset of overt ataxia and PC morphological alterations (at 12 weeks of age; Clark et al., 1997; Zu et al., 2004). Interestingly, restoration of the mGluR1-mediated synaptic signaling by the GABA_B_ receptor agonist baclofen rescued the motor functions in 12-week- old SCA1^82Q^ mice, underlying the importance of mGluR1 in SCA1 pathology; a single cerebellar injection of baclofen improved the rotarod motor performance in SCA1 mice for about 1 week (Shuvaev et al., 2017).

Disruption of mGluR1 signaling has been also observed in PCs from SCA3 (carrying the mutant ataxin-3 gene) as well as staggerer mice (with the mutation in the gene encoding ROR-α), while the AMPA receptor-mediated synaptic transmission was still preserved in PCs of both phenotypes (Konno et al., 2014). Immunofluorescence staining showed that mGlu1 receptors were mislocalized to non-synaptic sites and aggregated in PC dendrites of SCA3 mice. Similarly, decreased mGlu1 function has been assessed in a mouse model of human SCA5, caused by mutation β-III spectrin, a cytoskeletal protein anchoring mGluR1 at the membrane (Armbrust et al., 2014). In these mice, the mutant spectrin protein alters the stabilization of mGluR1, leading to diffuse distribution of receptors on PC dendrites and mGluR1 dysfunction.

On the contrary, the mGluR1 response was unaffected in the genetic FGF14 KO mouse model of SCA27, while the AMPA-mediated currents were significantly reduced due to a decrease in presynaptic glutamate release (Tempia et al., 2015). In such case, the presynaptic deficit is not sufficient to alter post-synaptic mGluR1 function since upon repetitive stimulation the Fgf14 deficient synapses can release sufficient glutamate to ensure full activation of perisynaptic mGlu1 receptors.

Mutations that result in the *mGluR1 gain of function* and excessive mGluR1 signaling, have been also linked to ataxia. For example, in the SCA2 mouse, the expansion of polyQ in the ataxin-2 protein causes an increase of IP3-induced Ca^2+^ release from PC ER and higher intracellular Ca^2+^ concentrations (Liu et al., 2009). The elevated Ca^2+^ levels result in the potentiation of mGluR1-mediated signaling; buffering basal Ca^2+^ concentration at physiological levels in SCA2 PCs prevents the increase of mGluR1 function (Meera et al., 2017). In addition, it has been demonstrated that SCA2 mice treated with inhibitors of IP3-induced Ca^2+^ release show reduced PC degeneration and improved motor coordination, suggesting that calcium dysregulation plays an important role in the pathogenesis of SCA2 (Kasumu et al., 2012).

Increased mGluR1 signaling has been also revealed in the PCs of *moonwalker* mice, carrying a mutation in the Trpc3 gene (Becker, 2014). This mutation leads to altered channel gating that promotes increased mGluR1-dependent inward currents and eventually excessive Ca^2+^ influx that may disturb the PC dendritic development. Indeed, in contrast to Trpc3 KO mice, the *moonwalker* mice exhibit a significantly impaired PC dendritic growth and arborization (Becker et al., 2009; Gugger et al., 2012). Mutation of PKCγ in SCA14 can also enhance TRPC3 currents and increase the amplitude of mGluR1-mediated slow EPSCs. The mutant PKCγ fails to phosphorylate and inhibit TRPC3 activity leading to sustained high levels of intracellular Ca^2+^, that may be responsible for the neurodegeneration characteristic of SCA14 (Adachi et al., 2008; Shuvaev et al., 2011).

## Ataxia Due to Alterations of the Climbing-Fiber/Purkinje Cell Synapse

Alteration of CFs architecture is a hallmark of some forms of SCA and other ataxias (Kano et al., 1997; Ebner et al., 2013; Smeets and Verbeek, 2016). CFs originate from the inferior olive of the medulla and make strong excitatory synapses onto the proximal domain of PC dendrites. At birth, PCs are normally innervated by multiple CFs, which undergo an activity-dependent refinement, such that by the end of the third postnatal week, most PCs are contacted by a single CF along their proximal dendrites (Lohof et al., 1996; Hashimoto and Kano, 2013).

In the early phase of CFs pruning, stronger CFs can activate postsynaptic Ca_V_2 channels more effectively than weaker CFs; the increase of postsynaptic Ca^2+^ levels will trigger LTP exclusively at large CF synaptic inputs (Bosman et al., 2008). LTP may further strengthen the already large synapses and allow formation of new synaptic contacts on the growing dendritic tree. Indeed, mice with a selective deletion of Ca_V_2.1 in PCs show persistent somatic innervation by multiple CFs associated with ataxic phenotype (see above; Miyazaki et al., 2004). According to studies *in vitro*, Ca^2+^ influx through Ca_V_ channels may activate Ca^2+^-dependent genes, such as Arc/Arg3.1, that are involved in synapse development, maturation and refinement (Mikuni et al., 2013). Perturbation of the pruning process and motor incoordination have been also observed in transgenic mice expressing a recombinant chloride channel specifically in PCs, that determines a genetic suppression of PC excitability (Lorenzetto et al., 2009).

The elimination of supernumerary CF synapses is significantly correlated with the development of PF synapses through a heterosynaptic competition (Scelfo and Strata, 2005; Hoxha et al., 2017). Indeed, in mutant mice that lack granule cells or functional PFs, such as *weaver*, *reeler* and *staggerer* as well as X-irradiated mice, the transition from multiple to single CF innervation is defective so that multiple innervation of PCs by CFs persists into the adult stage and is associated with ataxia and loss of motor coordination (Crepel et al., 1980; Crepel, 1982; Hashimoto and Kano, 2013).

Studies on mutant mice have also allowed the identification of several molecules involved in the late phase of CF synapse elimination, such as GluD2, mGlu1 and its downstream signal transduction pathway, including G_αq_, phospholipase Cβ4 and PKCγ (Chen et al., 1995; Offermanns et al., 1997; Kano et al., 1998). Mice lacking one of these mGlu1 signaling pathway components exhibit multiple CF innervation of PCs even in adulthood and motor discoordination. As reported above, the knock-out mice of the GluD2 receptor exhibit incomplete PF synaptogenesis, defects in the elimination of surplus CFs and impairment in LTD induction accompanied by alterations of motor coordination and motor learning. In these mice, CFs give rise to aberrant branches that not only extend distally to spiny branchlets (normally innervated by PFs) but also form ectopic synapses on adjacent spiny branchlets (Ichikawa et al., 2002). These results suggest that GluRD2 plays a critical role in shaping the compartmentalized innervation by CFs and PFs, restricting CF innervation to the proximal dendritic domain of the target PC and ensuring PF synapse formation on distal dendrites.

Elimination of redundant CF-PC synapses in the developing cerebellum is also regulated by retrograde signals from postsynaptic cells, such as the secreted semaphorin3A and the membrane-anchored semaphorin7A (Uesaka et al., 2014). The knockdown of Sema3A in PCs or its receptor, plexinA4, accelerated CF synapse elimination; whereas knockdown of Sema7A or its receptors in CFs, either plexinC1 or integrinB1, reduces CF synapse elimination (Uesaka et al., 2014). A recent study points out that BDNF derived from PCs can also facilitate elimination of CF synapses after P16 by binding to the B-type tyrosine kinase receptor (TrkB) on CFs (Choo et al., 2017). Similar to Sema7A, BDNF functions along the signaling cascade of mGlu1 since the effect of PC mGlu1 knockdown on the CF synapse elimination is occluded in BDNF-KO PCs.

Significant CF deficits have been also observed in mouse models of SCA1, SCA7, SCA14 and SCA23 (Ebner et al., 2013; Smeets and Verbeek, 2016). In particular, studies in SCA1^82Q^ mice have demonstrated that mutant ATXN1 preferentially affects CF–PC synapses in the early stage of disease, at 6 weeks of age, whereas alterations in PF–PC synaptic transmission did not occur until 28–40 weeks of age (late-stage disease; Barnes et al., 2011). Immunostaining of vesicular glutamate transporter type 2 (VGLUT2) revealed an abnormal CF terminal placement along the PC dendrites, that improved when mutant transgene expression was prevented during the second and third postnatal week. Interestingly, a severe form of ataxia and signs of motor deficit have been shown in the mouse mutant Ptf1a::cre;Robo3^lox/^^lox^, in which CFs derived from the inferior olive are rerouted so that they contact PCs located in the ipsilateral cerebellum, rather than the contralateral side (Badura et al., 2013). The motor performance of these mice appears to be even worse than that of the *Lurcher* mice (Van Alphen et al., 2002), suggesting that rewiring of the CF projection causes more severe ataxia than the loss of cerebellar cortex output.

## Conclusion

The great variety of mutations causing ataxia reveals the central role of PCs in the pathogenesis of motor symptoms in cerebellar diseases. Almost all ion channels expressed by PCs, when mutated, cause ataxic symptoms, whether Na_V_, Ca_V_, Kv, BK or regulatory proteins like Na_V_β4, Ca_V_β4, Ca_V_α2δ, Fgf14, Rer1 (Figure 1). This can be understood in the light of the complexity of ionic mechanisms necessary to PCs to carry out their computational tasks and generate appropriate output signals that, via deep cerebellar nuclei, allow a precise and fluent execution of movements and other brain functions.

Alterations of any synaptic input to the PC leads to cerebellar symptoms. In the case of the PF synapse, a deficit in the formation or stabilization, as in GluD2 or mGlu1 receptor mutants, is sufficient to cause a few motor symptoms related to ataxia (Figure 2). In contrast, even a slight disturbance of the maturation process of the CF-PC synapse can lead to a disruption of the innervation pattern, causing severe motor deficits. A further degree of complexity arises from the plasticity due to interaction between these two synapses.

Animal models with impairment of intrinsic membrane properties and those with synaptic deficiencies have in common an alteration of Purkinje cell output signals, independently from the mechanism involved. However, almost all reports about PC signaling in models of ataxia regard either spontaneous activity (spontaneous firing or spontaneous postsynaptic currents) or evoked responses to electrical or optogenetic stimulation (evoked firing or evoked postsynaptic potentials). This fact imposes serious limitations to the understanding of the mechanisms of motor control in ataxia. Future studies concerning the alterations of cerebellar output signals during the performance of motor tasks would give better insight into the specific mechanisms of motor control failure in the different types of ataxia.

## Author Contributions

MCM and FT designed the outline of the article. EH, IB, MCM and FT wrote the manuscript. EH and IB prepared the figures.

## Conflict of Interest Statement

The authors declare that the research was conducted in the absence of any commercial or financial relationships that could be construed as a potential conflict of interest.
